# Making Things Easier: A Simple Novel Method to Fix a Dorsiflexion Osteotomy of the First Metatarsal

**DOI:** 10.25122/jml-2019-0109

**Published:** 2020

**Authors:** Langhit Kurar, William Nash, Radwane Faroug, Laila Hussain, Roland Walker, Ali Abbasian, Ahmed Latif, Samrendu Singh

**Affiliations:** Department of Orthopedics, Guy’s and St Thomas’ NHS Foundation Trust, London, United Kingdom

**Keywords:** Foot, cavovarus, osteotomy, dorsiflexion, metatarsal, staple

## Abstract

A first ray dorsiflexion osteotomy is commonly performed for cavovarus foot correction. There are multiple techniques to fix this osteotomy, ranging from wires, screws, and plates or a combination of these. We present our results using a varisation staple (Biomet©) as an alternative fixation device. We performed a retrospective outcome analysis of a consecutive series of 10 cavovarus feet that underwent a dorsiflexion osteotomy (dorsal closing wedge) of the first metatarsal fixed with two varisation staples. The results were measured at a mean three monthly follow-ups and included union and complication rates, as well as clinical and radiographic assessment of cavus deformity correction. There was a 100% union rate with no complications or cases of delayed union. No metalwork removal was requested in any case at follow-up. First ray dorsiflexion osteotomies are most commonly fixed using a 3.5mm cortical screw. We demonstrate that our alternative and novel technique using varisation staples achieved a 100% union rate while avoiding the prominent hardware complications known to occur with cortical screws or plates.

## Introduction

First ray dorsiflexion osteotomies play an essential role in the surgical correction of the cavovarus foot [1, 2]. Swanson et al. first described dorsiflexion osteotomies in 34 patients in 1966 [3]. Since its first description, dorsiflexion osteotomies have been performed for cavovarus foot reconstructive surgery when there is residual first ray plantarflexion following tendon transfers.

There is no “gold-standard” technique to fix dorsiflexion osteotomies of the first metatarsal. A range of fixation methods such as K-wires, pins, cortical screws, and dorsal plates have been described [4-6]. Currently, the most commonly used fixation device is the cortical screw. However, delayed union and mal-union rates as high as 5% have been quoted amongst fixation methods [7-9]. Furthermore, when a cortical screw is used, care must be taken to countersink the head to avoid a dorsal cortical fracture. Utilizing a single screw can also compromise rotational stability and further increase the risk of non-union. Although the metalwork is usually well tolerated, it occasionally has to be removed due to irritation of the extensor tendons or skin surface [10].

This study aims to present our outcomes using a novel fixation technique for basal dorsiflexion osteotomy of the first metatarsal with a varisation staple.

## Material and Methods

Patients were identified using the hospital electronic database from the time of its introduction in 2008. Between February 2013 and June 2015, 10 cavovarus foot deformity cases (9 patients) underwent a dorsiflexion osteotomy of the first metatarsal that was fixed with a varisation staple. The average follow-up was three months, with a range of 6 weeks to 1 year. The wide range was mostly attributable to isolate cases where multiple procedures were conducted on the same foot to correct the deformity. Rehabilitation was subsequently prolonged. At follow-up, the patients were assessed clinically and with weight-bearing radiographs at six weeks to monitor union.

### The operative technique

The technique is performed under general anesthesia. The lower limb is exsanguinated, and a thigh tourniquet is applied and inflated to 300 mmHg. The limb is prepped and draped appropriately. The dorsiflexion osteotomy is commonly performed as part of a series of procedures to correct the cavovarus foot and can also be used in isolation.

A longitudinal incision is made, starting level with the first tarsometatarsal joint (TMTJ) and coursing distally along the shaft by 2-3 cm. The skin and subcutaneous tissues are incised and retracted, and any superficial veins encountered are cauterized. Extensor hallucis longus is protected and retracted laterally, and care is taken to respect the neurovascular bundle.

The osteotomy is marked at a distance of 10 mm from the tarsometatarsal joint. It is ensured that the obliquity of the metatarsal is appreciated, and the osteotomy is marked perpendicular to the shaft of the bone. A 10 mm long saw blade is used to perform the osteotomy. A perpendicular proximal cut is performed parallel to the first TMTJ, in the sagittal and coronal planes, but the preservation of the plantar cortex is ensured. The distal cut is made obliquely to join the proximal cut’s deep extent and thus complete the wedge resection. Again, care is taken to preserve the hinge of the plantar cortex. The thickness of the wedge depends on the degree of correction required. However, 3-5 mm is usually resected, and the osteotomy can then be closed without difficulty. A larger wedge may result in a degree of translation of the bone fragments or fracture of the plantar cortex. If the plantar cortex is inadvertently fractured, we would recommend plate osteosynthesis of the first ray instead.

Following the dorsal wedge resection, an 8 or 10 mm varisation staple is mounted on the holder. Our practice is to “paint” the staple tips using a skin marker so that the position of the pilot holes can be accurately marked on the bone. The osteotomy is then held closed, and the location of the pilot holes marked on the bone using the “painted” staple. Care is taken to place the proximal hole sufficiently distal to the TMTJ to avoid intra-articular penetration.

We use at least two staples and position these in the center of the dorsomedial and dorsolateral quarters of the metatarsal shaft, respectively. A third staple may be used between the two if necessary. A fine 1.1 mm wire is used to drill pilot holes for each limb of the staples, using the marks created in the previous step. With the osteotomy held closed, the staples are driven into the holes and gently “tapped home” using the staple punch.

Fluoroscopy is used to confirm the position of the hardware, and the wound is irrigated with normal saline solution and closed in layers.

**Figure 1:**
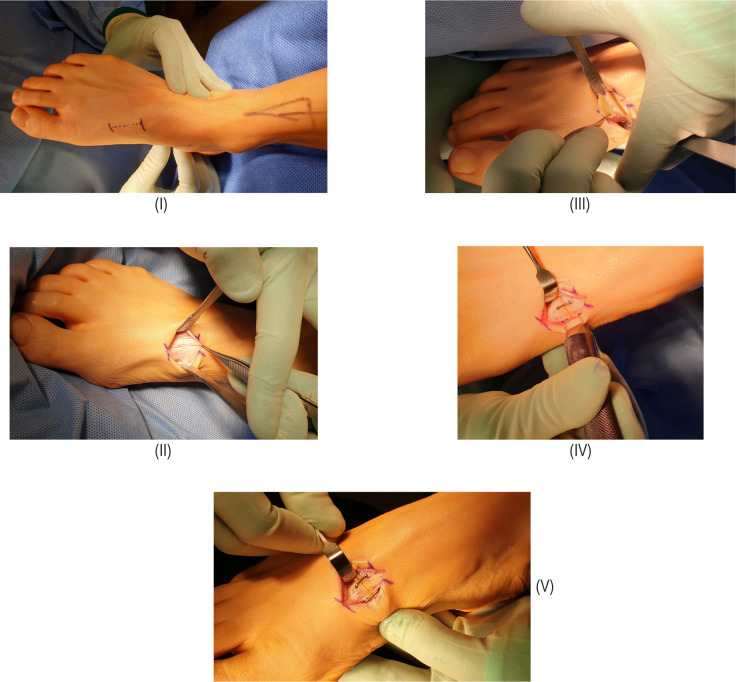
(I) Marked incision; (II) Layered dissection; (III) Saw cut with wedge osteotomy demonstrated; (IV) Application of staple; (V) Final result.

**Figure 2:**
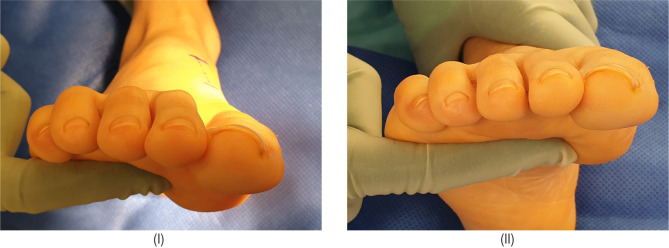
(I) Preoperative image demonstrating a plantarflexed first ray; (II) Postoperative correction.

**Figure 3:**
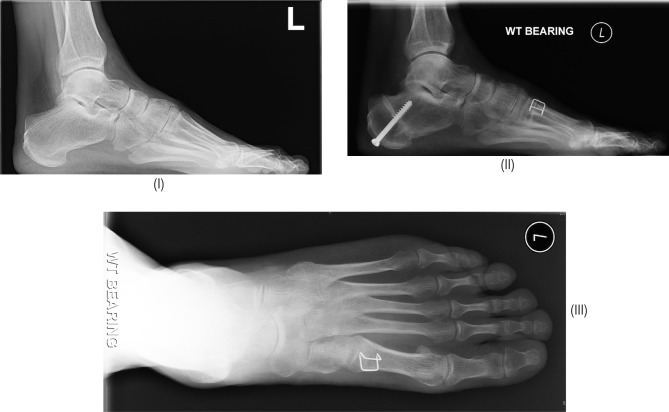
(I) Preoperative x-ray; (II) lateral weight-bearing x-ray showing healing of the basal first metatarsal osteotomy; (III) Antero-posterior weight-bearing radiograph.

Figure 1 demonstrates the step by step intra-operative technique to achieve the dorsal wedge osteotomy. Figure 2 demonstrates the degree of first ray dorsiflexion achieved. Preoperative and postoperative radiographs and the angular correction obtained are shown in Figure 3.

## Results

### Union

Union was defined as being pain-free with no movement at the osteotomy site on stress testing. Radiographic union was defined as forming bone trabeculae across the osteotomy site. All patients achieved clinical and radiological union by six weeks.

### Complications

We observed no complications in our series. Furthermore, there were no cases of hardware-related symptoms, and no removal was performed.

## Discussion

A first ray dorsiflexion is commonly performed as part of a series of procedures during cavovarus foot reconstruction. Since its first description in the 1960s, surgeons have used a variety of fixation techniques. Watanabe, in 1990, described a series of 50 feet that underwent dorsiflexion osteotomies. In these series, a Steinmann pin was used to stabilize the osteotomy. Although this technique yielded high union rates, the Steinmann pin had to be removed around five weeks later [8]. In 2001, Sammarco presented a series of 15 patients who underwent dorsiflexion osteotomies of the first metatarsal fixated using K-wires. Complications included delayed union in two and nonunion in three of the 66 metatarsal osteotomies, with a further procedure required to remove metalwork.

More recently, authors have used 3.5 mm cortical screws as their fixation device [11]. When performing a screw fixation, the surgeon must be careful not to countersink the screw head to avoid a fracture of the dorsal cortex. In our technique using a staple, this risk is avoided entirely, making the procedure technically easier.

In our consecutive case series, all our patients had a dorsiflexion osteotomy of the first metatarsal fixed using a varisation staple. All patients achieved full union both clinically and radiographically by 6 weeks follow-up. In other osteotomies of the first metatarsal, screw prominence is a recognized issue leading to skin irritation and hardware removal [12]. In our case series using staples, we report no complications associated with prominent hardware.

The cost per varisation staple is £21.15. A 3.5mm cortical screw costs £9.67, and a low profile locking plate costs £170. A cortical screw is the cheapest, but the varisation staple has the advantage of being less likely to require a second theatre episode for hardware removal, has a lower complication rate such as the risk of fracture, and is technically easier to perform with a lower tourniquet time.

To the best of our knowledge, this is the first presentation of the use of the “staple technique” as a fixation device for a first ray dorsiflexion osteotomy. The numbers included in this case series are small, and therefore, a larger population study is required to confirm and validate our findings. By and large, isolated first metatarsal dorsiflexion osteotomies were reserved for patients with a correctable hindfoot valgus, with the majority of the pressure symptoms distributed in the forefoot. Adjunct procedures, including a fifth ray procedure, were not required and certainly did not influence the study’s functional outcome [13].

## Conclusion

First ray dorsiflexion osteotomies are commonly fixated using a 3.5 mm cortical screw. Our study presents a novel fixation technique using a “varisation staple” to reduce complication rates and prominence of hardware requiring further surgery. In our case series, all patients achieved full union with no complications after a mean follow-up of 3 months.

## Conflict of Interest

The authors declare that there is no conflict of interest.
